# DNA Methylation Targeting: The DNMT/HMT Crosstalk Challenge

**DOI:** 10.3390/biom7010003

**Published:** 2017-01-05

**Authors:** Omar Castillo-Aguilera, Patrick Depreux, Ludovic Halby, Paola B. Arimondo, Laurence Goossens

**Affiliations:** 1 Univ. Lille, ICPAL, EA 7365—GRITA—Groupe de Recherche sur les formes Injectables et les Technologies Associées, 3 rue du Pr. Laguesse, F-59000 Lille, France; omar.castilloaguilera@univ-lille2.fr (O.C.-A.); patrick.depreux@univ-lille2.fr (P.D.); 2 FRE3600 Epigenetic Targeting of Cancer, CNRS, 31035 Toulouse, France; ludovic.halby@etac.cnrs.fr; 3 Churchill College, Cambridge CB3 0DS, UK

**Keywords:** DNA methylation, histone methylation, DNMT/HMT crosstalk, DNMT inhibitors, HMT inhibitors

## Abstract

Chromatin can adopt a decondensed state linked to gene transcription (euchromatin) and a condensed state linked to transcriptional repression (heterochromatin). These states are controlled by epigenetic modulators that are active on either the DNA or the histones and are tightly associated to each other. Methylation of both DNA and histones is involved in either the activation or silencing of genes and their crosstalk. Since DNA/histone methylation patterns are altered in cancers, molecules that target these modifications are interesting therapeutic tools. We present herein a vast panel of DNA methyltransferase inhibitors classified according to their mechanism, as well as selected histone methyltransferase inhibitors sharing a common mode of action.

## 1. Introduction

In humans, DNA methylation is a stable epigenetic mark that occurs at the C5 position of cytosines, mainly in a CpG dinucleotide context, but also in non-CpG regions of stem cells [1,2]. More than 50% of genes are associated with CpG islands in their promoter regions. Generally, low levels or a lack of DNA methylation in the promoter region is correlated with an “on” configuration of chromatin that favors the interaction of DNA with transcription complexes leading to the activation of gene expression. By contrast, methylation of CpG islands in gene promoters is correlated with an “off” configuration of chromatin that leads to gene silencing [3]. DNA methylation can maintain differential gene expression patterns in a tissue-specific and developmental-stage-specific manner. The roles of DNA methylation in gene bodies and other regions started to be characterized in the last years.

Three DNA methyltransferases (DNMTs), DNMT1, DNMT3a and DNMT3b, catalyze the transfer of a methyl group from *S*-adenosyl-L-methionine (SAM or AdoMet) to the C5 position of cytosine [4]. DNMT1 is responsible for methylating hemimethylated DNA and thus DNA methylation maintenance, whereas DNMT3a and DNMT3b are involved in de novo DNA methylation, but they can also participate in methylation maintenance. DNMT3a has two different isoforms, and DNMT3b has more than 30 isoforms [5,6]. They share common features: a well-conserved *C*-terminal domain bearing the catalytic activity. It contains motifs I to X, responsible for binding the SAM cofactor and the targeted cytosine. They also share an *N*-terminal domain that contains the ADD (ATRX-DNMT3-DNMT3L) motif and the nucleosome recognition PWWP (Pro-Trp-Trp-Pro) motif. These motifs are responsible for the interaction with DNA and with proteins that guide the enzymes to the nucleus, the chromatin and the DNA [2,7,8]. DNMT3L does not show a catalytic activity as it lacks the catalytic domain. It works as a coactivator of DNMT3a and DNMT3b, and is involved in the interaction with chromatin actors, improving methyltransferase activity by approximately 1.3 to 4 times [5,9]. It has been demonstrated that DNA methylation is correlated with histone-modification patterns [1,7].

Histones are subject to different post-translational modifications (acetylation, phosphorylation, ubiquitinylation, sumoylation, methylation, etc.) that control the expression of genes [10]. Histone methylation is, like DNA methylation, one of the most studied epigenetic modifications on histones. It can be linked to active transcription (e.g., H3K4me1/me2/m3, HK36me3, H3K79me1/me2/me3, H4R3me1, H4K20me1) or to gene silencing (e.g., H3K9me2/me3, H3K27me3) [10,11]. Histone methyltransferases (HMT) use, as DNMTs, the SAM cofactor to mono-, di- or tri-methylate lysine residues (thus known as HKMT, histone lysine methyltransferases) or to mono- or di-methylate arginine residues (thus known as PRMT, protein arginine *N*-methyltransferases) of the core or tails of histones [12]. The location and the level of methylation of histones can differently influence DNMT activity. DNA and histone methylation show an important crosstalk [1,13]. For example, the trimethylated residue lysine 36 of the tail of histone 3 (H3K36me3) is linked to gene body DNA methylation and it can be read by the PWWP domain of DNMT3a and DNMT3b to guide DNA methylation. In consequence, a mutation of the PWWP domain or the absence of the epigenetic mark H3K36me3 causes the loss of DNMT–nucleosome interaction, leading to decrease of DNA methylation at pericentromeric regions [7,14]. In contrast, at promoter regions, the unmodified H3 can be recognized by DNMT3 via the ADD domain and H3K4 methylation inhibits DNMT3 activity [1].

DNA methylation has an essential role for cell differentiation and development. However, the DNA methylation profile can be altered leading to DNA instability and triggering diseases such as cancer [12,15,16]. In tumorigenesis, methylation in the promoter regions of some genes—such as tumor suppressor genes (TSGs) involved in cellular cycle (e.g., cyclin-dependent kinase (*CDK*) inhibitors, retinoblastoma protein (*RB*)), maintenance of genome integrity (e.g., *TP53*, breast cancer 1 (*BRCA1*), O^6^-methylguanine DNA methyltransferase (*06-MGMT*), mutL homolog 1 (*hMLH1*)), apoptosis (e.g., *caspase 8,* death-associated protein kinase (*DAPK*), migration process (e.g., E-cadherin (*CDH1*), metalloproteinase inhibitor 3 (*TIMP-3*)), and those involved in the response to growth factors (phosphatase and tensin homolog (*PTEN*), estrogen receptor (*ER*)) leads to their silencing. At the same time, low levels of gene body methylation participate in genome instability [17,18]. Therefore, inhibition of DNA methylation is an interesting approach in cancer treatment [19,20,21,22]. 

## 2. Inhibition of DNA Methylation

### 2.1. Cytidine Analogs

**Figure 1:**
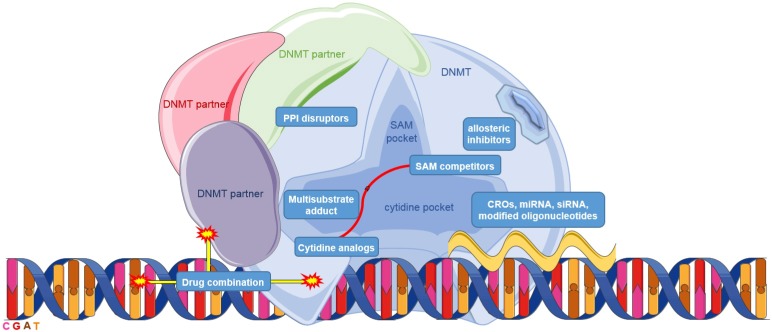
Schematic representation of different DNA methyltransferase (DNMT)-inhibition approaches. DNMT: DNA methyltransferase; PPI: protein–protein interaction; SAM: *S*-adenosyl-L-methionine; CRO: chimeric RNA oligonucleotides; miRNA: micro RNA; siRNA: small interfering RNA.

**Figure 2:**
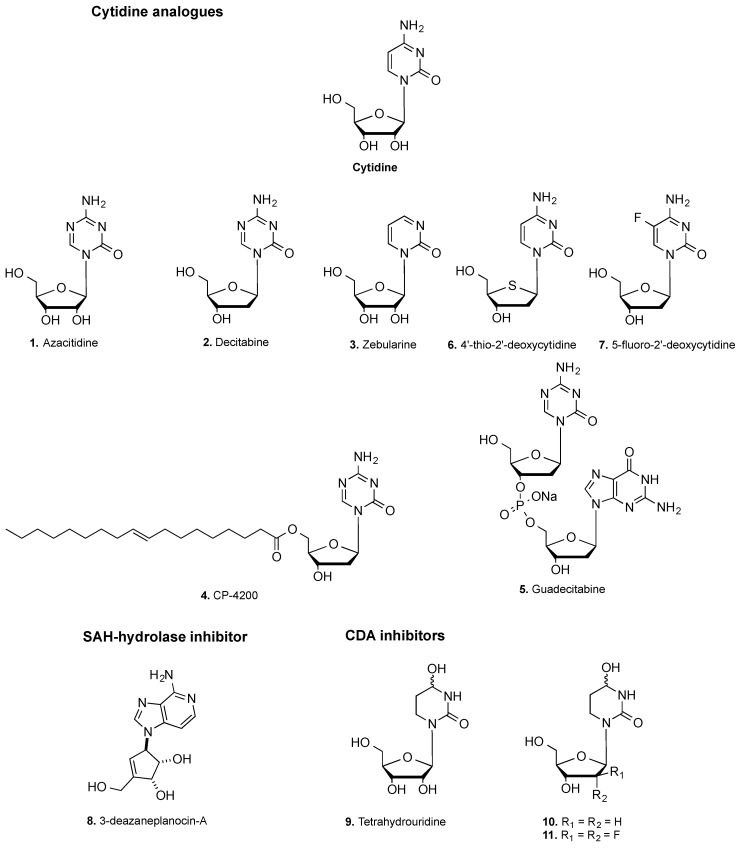
Structures of nucleoside DNMT, *S*-adenosyl-L-homocysteine (SAH)-hydrolase and cytidine deaminase (CDA) inhibitors.

Up to date, several strategies to inhibit DNA methylation have been developed (Figure 1). At present, a suicide substrate of DNMTs is the most advanced approach. 5-Azacytidine or azacitidine (**1**) and 5-aza-2′-deoxycytidine or decitabine (**2**) (Figure 2) are indeed the only two DNMT inhibitors (DNMTi) approved by the USA Food and Drug Administration (FDA) and the European Medicines Agency (EMA) for the treatment of acute myeloid leukemia (AML), chronic myelomonocytic leukemia (CMML) and myelodysplastic syndromes (MDS) [17]. These nucleoside analogs, i.e., cytidine analogs, incorporate DNA instead of deoxycytidine, covalently link the enzyme and lead to DNMT degradation [23,24]. Although these molecules are particularly active, they have poor chemical and metabolic stability, low specificity—since they incorporate into DNA replacing all cytidines—and they induce several side effects [2]. A more stable and less toxic cytidine analog, zebularine (**3**), was developed [25,26]. However, its efficacy at very high doses prevented it to enter the clinical trials. Given the success of (**1**) and (**2**), prodrugs of these molecules, for instance CP-4200 (**4**), an elaidic acid ester analog of (**1**), and SGI-110 (or guadecitabine) (**5**), a dinucleotide decitabine-*p*-deoxyguanosine, were developed to improve the pharmacokinetic profile [27,28]. The latter compound is in clinical trials for the treatment of AML, MDS, ovarian and liver cancers (NCT01261312, NCT02901899, NCT01752933).

4′-Thio-2′-deoxycytidine (TdCyd) (**6**) is another cytosine analog in clinical trials for patients with advanced solid tumors (NCT02423057). This compound incorporates into the DNA sequence recognized by the bacterial C5 DNA methyltransferase M.HhaI, leading to DNA methylation inhibition. Furthermore , it depletes DNMT1 in both in vitro and in vivo cancer models [29,30]. 5-fluoro-2′-deoxycytidine (FdCyd) (**7**) has been enrolled in clinical trials for treatment of advanced solid tumors, AML and MS ( NCT00359606, NCT01041443) [31].

Recently, preclinical studies have shown that (**1**) in combination with 3-deazaneplanocin-A (DZNep) (**8**) produce a synergistic reactivation of *CDKN1A* (Cyclin-dependent kinase inhibitor 1A), *CDKN1B* and *FBXO32* (F-Box Protein 32) genes [32,33]. In fact, (**8**) is an inhibitor of *S*-adenosyl-L-homocysteine (SAH) hydrolase, an enzyme involved in the degradation of SAH (or AdoHcy), the product of the methylation reaction and a natural inhibitor of HMTs and other enzymes. In addition, (**1**), (**2**), and (**5**) are in clinical trials in combination with other drugs (i.e., cytotoxics, monoclonal antibodies or HDACi) to improve the effects and to reduce side effects of monotherapy [34]. To increase their potency, cytidine analogs have been studied in combination with inhibitors of cytidine deaminase (CDA), an enzyme mainly found in the gastrointestinal tract and liver involved in their inactivation by deamination, limiting their bioavailability. These compounds are tetrahydrouridine (THU) (**9**) or its improved deoxy- and difluorinated derivatives (**10** and **11**) [35]. Compound E7727 (structure not yet disclosed) is a CDA inhibitor tested in clinical trials (NCT02103478) in a combined formulation with (**2**) under the name ASTX727.

To overcome the non-specificity of the nucleoside inhibitors, several non-nucleoside molecules have been developed in the past years. Their structures are very heterogeneous, but for all of them their mechanism of action is independent of DNA incorporation. Thus, this class has drawn special attention.

### 2.2. DNA Binders

**Figure 3:**
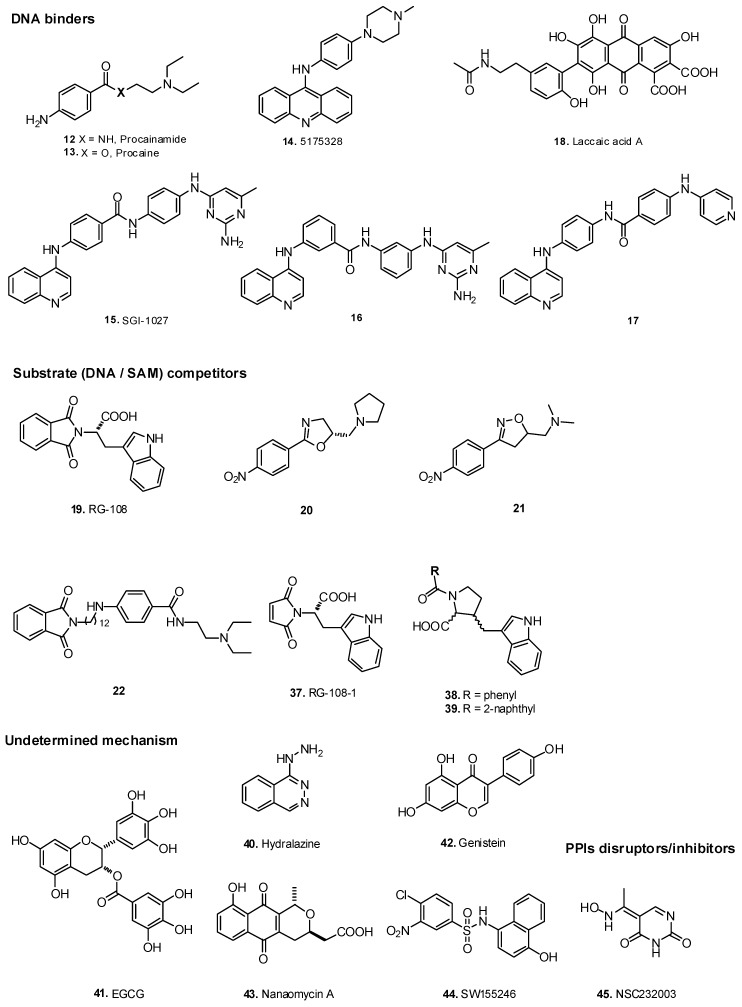
Structures of non-nucleoside DNMT inhibitors.

Some commercial drugs have been repurposed as they were discovered to show demethylating effects, such as amide procainamide, an antiarrhythmic drug (**12**) and its ester analog procaine, a local anesthetic (**13**) (Figure 3, Table 1). These molecules showed affinity for CpG-rich regions of DNA blocking the activity of DNMTs and reactivating some TSGs [36,37,38]. Other DNA binders tested for DNA methylation inhibition are derivatives of acridine, a heterotricycle known to intercalate into DNA. Particularly, compound 5175328 (**14**) was able to reactivate methylated silenced genes. Unlike nucleoside analogs (**1**) and (**2**) that inhibit DNMT after at least two cell division rounds, as they need to incorporate in DNA, acridine derivatives showed demethylation activity after only one cell division round [39]. However, as DNA binders, these compounds can interfere with other DNA enzymes and need to be improved for selectivity.

Compound SGI-1027 (**15**), a quinolone derivative previously considered as a SAM cofactor competitor, has been recently proven to inhibit DNMT1 and DNMT3a by a DNA-binding mechanism [40,41]. It was also proven to cause DNMT1 proteosomal degradation in colon cancer cells and to demethylate and reactivate *TIMP3*, *MLH1* and *P16* promoters in HCT116 cells [41]. Due to the positive results of this compound, structure–activity relationship (SAR) studies have been performed to improve the activity of (**15**). Consequently, derivatives (**16**) and (**17**) also showed a DNA-competitive inhibition of DNMT. Compound (**16**) is the most potent DNMT1 inhibitor among them [4,42,43]. 

Finally, a natural product, the highly substituted anthraquinone, laccaic acid A (**18**), was described as a direct, DNA competitive inhibitor of DNMT3a and M.SssI methyltransferase with moderate selectivity for DNMT1. It was also shown to reactivate methylated TSGs [44].

Although DNA competitive or non-competitive binders have shown a particular interest as DNMT inhibitors and TSG reactivators, it is important to highlight that they need CpG-region selectivity at hypermethylated TSGs in cancers in order to not unspecifically affect proteins that recognize and bind DNA. 

### 2.3. Oligonucleotides

Besides DNA binders, short RNA molecules (4–8 nucleotides) are theoretically long enough to be accommodated in the catalytic pocket of DNMTs and to be effective, competitive inhibitors. With this aim, chimeric RNA oligonucleotides (CROs) have been developed; they specifically target genes and reduce DNMT catalytic activity. The CROs can bind a carrier (e.g., lipopolysaccharide, liposome, nanoparticles) in a covalent or non-covalent way that favors its transport into a particular cell type. The CROs are formed by 15–30 nucleotides with one or two modified nucleotides. They are at least 80% complementary to a portion of an extracoding RNA of a gene. Once they bind, the complex form binds DNMT and prevents DNA methylation of this gene [20,54]. Other small RNAs have also been studied as DNA competitive inhibitors of DNMTs. Unlike the CROs previously described, New England Biolabs Inc. (Ipswich, Massachusetts, USA) identified small RNA molecules (Table 2, entries 1–3) that inhibit DNMT activity globally. Their complementarity to human genes is less than 80% [20,55]. Another type of oligonucleotide includes at least one modified CpG dinucleotide that functions to trap the DNMTs. On one strand, the cytosine of CpG is replaced by a cytosine analog -(**1**), (**2**), (**3**) of Figure 2, for instance, and, on the opposite strand, the cytosine remains unmodified or substituted by a methylated cytosine (to create a hemimethylated target for DNMTs). These oligonucleotides are configured to form a double-stranded hairpin when annealed (Table 2, entries 4–6) [20,56].

Other oligonucleotides, named epi-miRNAs, were developed to directly inhibit the transcription of DNMTs [60]. These micro RNAs are complementary to the 3′-untranslated region of the DNMT1 mRNA and lead to blocking DNMT gene transcription. Several mi-RNAs that directly target DNMT mRNA have been developed. For instance, oligonucleotide MG98 (Table 2, entry 7), a 20-nucleotide-antisense sequence with phosphorothioate linkages and 2′-*O*-methyl modifications, has shown interesting preclinical results proving inhibition of DNMT activity, re-expression of TSGs and tumor growth inhibition. Unfortunately, the clinical trials failed due to high toxicity and poor efficacy [57,58,61,62,63,64]. In order to improve the activity and to reduce toxicity, this oligonucleotide is the subject of further studies in combination with other agents [58]. Another example of epi-miRNA is the oligonucleotide miR29b (Table 2, entry 8) that targets DNMT1, DNMT3a and DNMT3b, leading to a decrease of DNA methylation levels and the re-expression of TSGs [59,65,66,67].

In parallel, small interfering RNAs (siRNAs), short, non-coding double-stranded RNAs, have been designed to directly target the genes that code for DNMT1, DNMT3a and DNMT3b. After DNMT degradation, they promote the expression of CTA (cancer/testis antigen), regulated by DNMTs that may be beneficial for immunotherapy of tumors [68,69]. However, their poor bioavailability and stability in physiological conditions limits their development in clinics.

### 2.4. S-adenosyl-l-methionine Cofactor Competitors

A very interesting DNMT inhibition approach is the targeting of SAM cofactor binding site in the enzyme. Several molecules have been described to be SAM competitors showing DNMT inhibition and leading to TSG reactivation. The first one was phthalimido-L-tryptophan RG-108 (**19**), identified as a hit in a virtual screening and validated as an inhibitor of the DNMT activity in vitro and in cancer cells. It was shown to reactivate TSGs, such as *P16*, *TIMP3* and *SFRP1*, in the colon cancer cell line HCT116 by promoter demethylation [70,71]. 

Chemical modifications of the DNA ligands (**12/13**) led to constricted oxazoline and izoxazoline derivatives (**20**) and (**21**) [48,72]. Unlike (**12/13**), these derivatives resulted as SAM competitors. Since they showed in vitro activity over DNMT1, inhibition of HCT116 cell proliferation and weak global DNA demethylation in cancer cells, they represent lead molecules to be further optimized.

Conjugates of (**12**) (a DNA binder) with (**19**) (a SAM competitor) have been reported (**22**) [45]. As expected, procainamide concentrated the conjugate at CpG-rich regions, while the RG-108 part was well positioned to inhibit the enzyme. Thus this strategy resulted in inhibitors up to 50 times more active than the parent compounds. They were also found to be selective for DNMTs versus mammalian histone G9a methyltransferase. 

Development of SAM competitors to inhibit DNMT represents a promising strategy. Nevertheless, since other methyltransferases use SAM as their cofactor, such as histone methyltransferases, DNMT specificity is a challenge. 

## 3. Inhibition of Histone Methylation

HMTs are a large family of protein methyltransferases (over 50 of them) that methylate lysine or arginine residues present in the core or in the tails of histones. Due to the direct or indirect role of some HMTs in tumorigenesis, HMT inhibitors (HMTi) have been developed [21,73]. Development of SAM competitors remains one of the most advanced approaches.

### 3.1. Histone Lysine Methyltransferases

**Figure 4:**
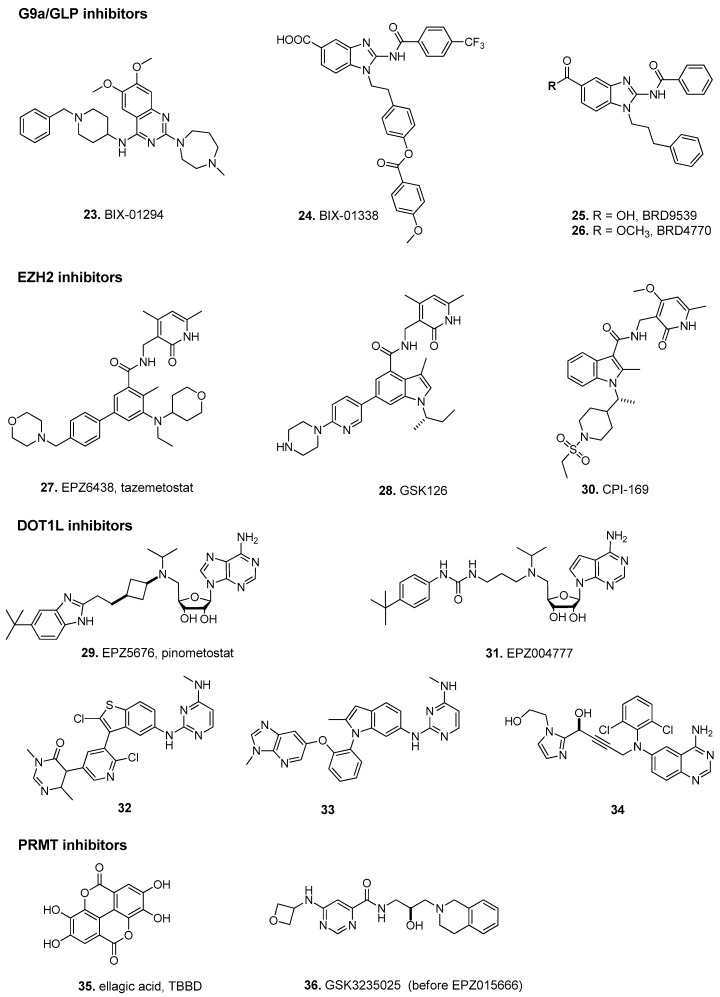
Structures of selected histone methyltransferases (HMT) inhibitors. G9a: euchromatic histone-lysine *N*-methyltransferase 2; GLP: G9a-like protein; EZH2: enhancer of zeste homolog 2; DOT1L: disruptor of telomeric silencing 1-like; PRMT: protein arginine *N*-methyltransferase.

All HKMT contain the conserved protein–protein domain SET (*Su(var)3–9*, *Enhancer of Zeste*, *Trithorax*), except for disruptor of telomeric silencing 1-like (DOT1L). Among the HKMT family, G9a and G9a-like protein (GLP) are two HKMTs that catalyze H3K9me1 and H3K9me2, and with Suv39H1, Suv39H2, and SETDB1, complete the SET-containing SUV39 protein family responsible for H3K9 methylation, an epigenetic modification found to be dependent on DNA methylation in human cancer cells [74,75,76]. G9a and GLP apparently form a functional heteromeric complex with in vivo H3K9 methyltransferase activity [76,77,78,79]. Furthermore, this complex was shown to promote tumor growth, affect cell cycle or metabolism pathways [80]. Several inhibitors have been developed, such as the substrate-competitive compound BIX-01294 (**23**) and the SAM-competitor compound BIX-01338 (**24**), both discovered in the same HTS (Figure 4, Table 3) [81]. To improve their selectivity and to decrease their toxicity, other compounds were synthetized [82], such as compound BRD9539 (**25**) and its methyl-ester analog BRD4770 (**26**), found to be useful as probes of G9a [83]. Compound (**25**) seems to be the active form of (**26**). The latter was active in cell-based assays and it was shown to reduce cellular levels of H3K9 methylation (without inducing apoptosis), to induce senescence, and to inhibit proliferation in the pancreatic cancer cell line PANC-1 [83]. In addition, other pharmacomodulations of (**23**) led to a quinazoline derivative that shifts G9a/GLP inhibition to DNMT3a inhibition, suggesting the interest in studying the DNMT/HMT specificity of DNMT inhibitors [84]. In mammals, DNA and H3K9 methylation are strongly associated. Indeed, DNA methylation is lost in *G9a* or *GLP*-mutated cells. G9a and GLP can recruit DNMT3a and DNMT3b directly or indirectly through the chromodomain protein M-phase phosphoprotein 8 (MPP8), leading to *de novo* DNA methylation [75,85,86,87,88]. 

Importantly, it was shown that it was possible to find selective SAM-mimetic molecules to inhibit HMTs potently enough to enter clinical trials [12]. For instance, EPZ6438 (tazemetostat) (**27**) [89], GSK126 (**28**) [90] and CPI-1205 [91], SAM-competitive inhibitors of Enhancer of Homolog Zeste 2 (EZH2), are currently in phase I/II clinical trials. Compound EPZ5676 (pinometostat) (**29**) [92,93,94], a SAM-competitive DOT1L inhibitor, has just completed phase I clinical trials (last updated August 2016), its clinical development was stopped due to the lack of efficacy in monotherapy. 

EZH2 and its homolog EZH1 are part of the core of the polycomb repressive complex 2 (PRC2). PRC1 and -2 are both involved in transcription repression of specific genes. EZH2/EZH1 are responsible for H3K27 methylation, which maintains transcriptional silencing. Ectopic expression of EZH2 is considered as a biomarker of metastasis and poor-prognosis tumors. EZH2 and DNMT have been suggested to have a cross-mechanism of epigenetic silencing that contributes to transcriptional repression of specific genes in cancer cells [95,96,97,98,99]. Hence, several inhibitors have been developed to specifically target EZH2. Compound CPI-169 (**30**) inhibits the catalytic activity of PRC2, decreases H3K27me3, and triggers cell cycle arrest and apoptosis in different cell lines. In combination with other drugs, it caused tumor regression in a KARPAS-422 model [91,100]. To date, three compounds are in clinical trials: (**27**) for B-cell and follicular lymphomas, sarcoma, mesothelioma and advanced solid tumor treatment (NCT01897571, NCT02601950, NCT02601937, NCT02860286); (**28**) for B-cell, follicular, and other non-Hodgkin’s lymphomas, solid tumors, and multiple myeloma (NCT02082977); and CPI-1205 for B-cell lymphoma treatment (NCT02395601). Compounds (**27**) and (**28**) contain a 2-pyridone moiety linked to a benzamide or indole-amide core, and they have a SAM-competitive mechanism. 

Compound (**29**) is the result of pharmacomodulations mimicking SAH. The adenosine derivative (**29**) and its phenylurea analog (**31**) (EPZ004777) [101] showed very good inhibition and specificity for DOT1L, reduced leukemia-relevant gene expression and induced differentiation of MLL (mixed-lineage leukemia) leukemia cells. DOT1L is the only non-SET domain HKMT and it is the only enzyme responsible for mono-, di- and trimethylation of H3K79, leading to transcriptional activation of certain oncogenes [102,103,104,105,106,107]. It is mainly involved in myeloid lymphoid leukemia with *MLL* rearrangements by favoring transcription of *HOX* (subset of homeotic genes) and *MEIS* (Meis homeobox 1) genes involved in acute leukemia development [105,106,108]. Therefore, medicinal chemistry efforts for DOT1L inhibition have led to the first HMTi in clinics, compound (**29**) that completed phase I clinical trials for leukemia treatment and myelodysplastic syndromes (NCT02141828, NCT01684150). In order to improve its pharmacokinetic properties, other SAH-mimetics were synthesized [109], non-ribose-containing analogs have been developed [110] and non-nucleoside derivatives have been obtained from a fragment-based approach. Novel structures have been disclosed such as (**32**), (**33**) and (**34**) that also show a SAM-competitive mechanism to inhibit DOT1L [111,112]. Other derivatives with IC_50_ in the micromolar ranges have been identified by means of docking screenings and in silico studies as well [113,114].

### 3.2. Protein Arginine N-Methyltransferases

The protein arginine methyltransferases (PRMT) are a family of 11 enzymes that catalyze mono- or dimethylation of arginine residues on histones. As HMTs, they use SAM as methyl donor. Up to date, PRMT inhibitors (PRMTi) are still limited to preclinical studies. Ellagic acid (TBBD) (**35**) and pyrazole-containing derivatives have been elucidated as inhibitors of coactivator-associated arginine methyltransferase (CARM1, also known as PRMT4), responsible for catalyzing H3R17me2 and H3R26me2, modifying non-histone proteins (e.g., p300/CBP (CREB-binding protein) and SRC-3 (Steroid receptor coactivator-3)), co-activating several transcription factors (e.g., steroid receptors) and being involved in prostate and breast cancer progression [117,119,120,121]. GSK3235025 (previously known as EPZ015666) (**36**) was proven to be a potent, selective inhibitor of PRMT5, a PRMT responsible for catalyzing H4R3me2 and H3R8me2, being active on non-histone substrates (e.g., p53, programmed cell death 4 (PDCD4)) and acting as a transcriptional repressor. PRMT5 deregulation has been linked to tumorigenesis [122,123] and (**36**) showed efficacy in in vitro and in vivo models of mantle cell lymphoma (MCL) [118,124]. Compound (**36**) was used as a probe for the enzyme study, while the improved compound GSK3326595 (previously known as EPZ015938), has recently entered dose escalation phase of clinical trials (NCT02783300) for the treatment of solid tumors and non-Hodgkin’s lymphoma [125]. The chemical structure of the latter compound has not yet been disclosed. CARM1-specific and PRMT5-specific inhibitors bind the substrate-binding pocket, rather than competing with the SAM cofactor. However, interestingly, the binding of SAM is needed for the activity of (**36**).

## 4. DNA Methyltransferase-Isoform Selectivity

An interesting but controversial issue is the selectivity towards DNMT isoforms. Selective compounds will allow studying the role of each isoform in different cancers and identifying the best DNMT isoform for target in cancer cells [17,126,127]. The studies on the HMT inhibitors and on the kinase inhibitors have illustrated that it is possible to design specific cofactor-mimicking inhibitors. Therefore, this might also be possible for DNMTs [128]. Although the catalytic pockets of DNMTs are well conserved, some amino acid residues are different. For instance, Trp1173 in DNMT1 is replaced by Cys662 in DNMT3a, Asn1580 by Arg887, and Val582 by Trp889. Thus, design of selective DNMT inhibitors could be achieved [17]. Also, it has been observed that the SAM cofactor can adopt a different conformation in its binding pocket according to the type of methyltransferase, which can provide a molecular basis for ligand-based design and pharmacophore-based screening to develop SAM-competitive inhibitors [129]. It is noteworthy that the catalytic pockets are dynamic, and inhibitors can induce conformational changes, as is the case for compounds (**29**) and (**31**) that bind in the the SAM cofactor binding site of DOT1L [93,130], thereby inducing a conformational change that leads to a gain of selectivity. 

## 5. Inhibition of DNA Methylation: Other Approaches

### 5.1. Allosteric and Bisubstrate Approaches

As other enzymes, DNMTs should have allosteric sites that can be targeted to regulate their activity. No compounds have been identified with this mechanism of action.

Since the methyltransferases have two substrates, the cofactor and the DNA, a multisubstrate approach can be considered.

Compounds such as maleimide derivatives (**37**) (also known as RG108-1), (**38**) and (**39**), developed from the SAM-competitive DNMT inhibitor (**19**), were shown to fit not only in the SAM cofactor pocket, but also in the cytidine binding pocket as suggested by an in silico model (Figure 3, Table 1) [47,46]. Some flavones and flavanones have been identified to inhibit DNMT3a/3L complex in lower micromolar ranges by a mixed mechanism according to docking studies [51].

The hybrids of (**12**) with (**19**) were designed considering this combined strategy, and (**22**) was elucidated with greater inhibition activity compared to the parent compounds [45].

### 5.2. Repositioned Drugs and Natural Products

As evoked above, certain commercial drugs showed demethylating effects. This was also the case of hydralazine (**40**), an antihypertensive drug that has led to reactivation of TSGs without causing a global genomic demethylation in cells [36,131]. The mechanism of action of hydrazaline is still a controversial issue as some groups claimed that it binds to the catalytic site of DNMT, while others reported that it reduces DNMT1 and DNMT3a expression via the extracellular signal–regulated kinase (ERK) pathway inhibition [132,133]. This drug is in different phases of clinical trials as an anticancer drug, and registered in Mexico in combination with an HDAC inhibitor, i.e., magnesium valproate, for MDS treatment [134,135].

In addition to repositioned drugs, several natural products have shown demethylating effects. For example, the natural polyphenol (−)-epigallocatechin 3-gallate (EGCG) (**41**) is proven to decrease DNA methylation and to reactivate the TSGs *P16*, *P21*, *MGMT*, *RARβ2* (retinoic acid receptor β2) in cancer cell lines [49,50,136,137]. However, its action mechanism has not been elucidated. It has been hypothesized that SAH production by the SAM-mediated methylation of EGCG, catalyzed by catechol-*O*-methyltransferase (COMT), leads to a negative feedback on SAM-dependent methyltransferases, such as DNMT, although in silico studies have suggested that (**41**) binds the catalytic site of the enzyme, leading to a direct DNMT inhibition [137,138] and DNMT3a degradation [139]. Most recently, the antiproliferative effect of (**41**) has been studied in the presence of COMT inhibitors (i.e., entacapone and tolcapone) supporting the idea that it is active by different pathways and targets [140]. As flavonoids are multi-target compounds [141], it is difficult to consider (**41**) or genistein (**42**), the main soybean constituent, as potential therapeutic tools. Nonetheless, synthetic flavonoids have been identified to show specific DNMT inhibition activity [51].

The natural quinone antibiotic nanaomycin A (**43**) [52,142] and a sulfonamide derivative identified in an HTS, SW155246 (**44**) [53,143], have garnered attention as they have shown DNMT inhibition activity and weak but considerable demethylation of promoter regions of specific genes, leading to TSG reactivation. Compound (**43**) seems to have other targets and its demethylation activity needs further validation.

Even if in silico studies have shown that the compounds cited in this section interact with the catalytic site of DNMT, further biological studies are needed to determine their real mechanism.

### 5.3. Protein-Protein Disruptors

Another approach to inhibit DNMT activity is to target the protein–protein interactions (PPI) needed for the interaction of the enzyme with its partners [8,20]. Earlier in this paper, two interactions were discussed: H3K36me3 recognized by the PWWP domain of DNMTs, and unmodified H3 recognized by the ADD domain of DNMT3a [1,7,14]. In the first case, the PWWP domain of DNMTs can be directly targeted, or the methylation of H3K36 can also be inhibited to indirectly affect DNA methylation. In fact, H3K36 has been proven to have an important role in tumorigenesis; several H3K36 methyltransferases and fusion partners of this modification have been found to be dysregulated or overexpressed in some cancers (i.e., nuclear SET domain containing proteins, ASH1 like histone lysine methyltransferase , SET and MYND domain containing 2 protein (SMYD2)) [144]. In the second case, the ADD domain of the enzyme can be targeted to disrupt the interaction with the nucleosome. It is known that methylated H3K4 disrupts the ADD–H3 interaction [145]. Also, another potential interacting site is between ubiquitin like with PHD and ring finger domains 1 protein (UHRF1) and DNMT1. UHRF1 is an accessory protein responsible for the interaction of the enzyme with the hemimethylated DNA [146]. This protein has different domains that bind to other marks, such as the tandem Tudor domain that binds H3K9me2/3 [147], and an ADD-like domain that binds the histone H3 tail [148]—an example of the DNA/histone methylation crosstalk. Although several PPIs have been described, no validated DNMT inhibitors have been identified following this strategy. Up to date, some studies have given interesting results, such as peptides that inhibit the DNMT1/CFP1 interaction, important for the enzyme functionality [149]. Most recently, a uracil derivative NSC232003 (**45**) has been found to inhibit in vitro DNA methylation by disrupting the DNMT1/UHRF1 interaction at a cellular level [150]. In addition, a clustered regularly interspaced palindromic repeat clustered regularly interspaced short palindromic repeats (CRISPR)/Cas9 system was proven to affect the DNA methylation pattern, and it may represent a novel profitable approach [151]. Disruption of PPIs in DNMT-involving complexes represents a very specific and targeted cancer treatment approach [8].

### 5.4. Multitarget Inhibitors

Given the crosstalk of DNA and histone methylation, a multitarget approach is a profitable challenge to develop new molecules with enhanced anticancer activity. As evoked above, the combination of DNMTi **1** and the SAH hydrolase inhibitor (**8**) represents this emerging approach [33]. Furthermore, keeping in mind that SAM is the cofactor of both DNMT and HMTs, dual inhibitors, i.e., agents with a double effect, might also represent an interesting anticancer approach [84].

## 6. Conclusions

**Figure 5:**
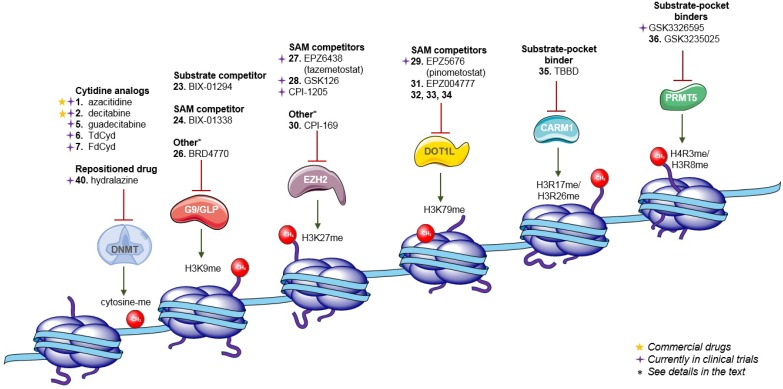
Summary of DNMT and HMT inhibitors. The molecules labeled with a star are commercial and those marked with a cross are currently in clinical trials.

The ongoing studies of DNA methylation, including its crosstalk with histone methylation and the protein complexes involved, have pointed out its role in cancers. Therefore, DNMTs are validated therapeutic targets, as proven by two commercial drugs ((**1**) and (**2**)) targeting DNA methylation. Clinical investigation on epigenetic agents that show only weak or no clinical activity should not be abandoned as some of these agents may produce a potent enhancement of the clinical activity of other epigenetic agents. Thus, clinical trials of these combinations should be investigated, especially the combinations that are supported by found preclinical data. In fact, clinical studies are ongoing using these epi-drugs in combination with cytotoxic agents, immune therapy or with other epi-drugs (histone deacetylase inhibitors, HDACi) (Figure 5) [152]. Several technological efforts have been used to discover new, less toxic DNMT inhibitors: high-throughput screening (HTS) of different rich libraries, docking-based virtual screenings, fragment-based design, molecular modelling from the enzyme crystal structures, optimization of current DNMT inhibitors and rational design of new ones. Despite the efforts to develop new DNMT inhibitors, this task remains a challenge, not only to have new drugs, but also to develop selective probes that contribute to the better understanding of the DNA methylation. The very promising results with molecules reprogramming cancer cells give hope to pursuing this task.

## Figures and Tables

**Table 1:** Non-nucleoside DNA methyltransferase inhibitors (DNMTi) and their activity.

Inhibitor	IC_50_ (or EC_50_) ^a^, µM			Reference
	DNMT1	DNMT3a	DNMT3b	
**12** (procainamide)	>500	>300 ^a^	ND	[38]
**15** (SGI-1027)	6	8	7.5	[40]
**16**	9	2.8 ^b^	ND	[4]
**17**	(15)	(0.9)	ND	[43]
**18** (laccaic acid A)	19	50	ND	[44]
**19** (RG-108)	390	315 ^b^	ND	[45,46]
**37** (RG108-1)	20	ND	ND	[47]
**38a** (2*S*, 3*R*)	98	ND	ND	[46]
**38b** (2*R*, 3*S*)	73	ND	ND	
**39a** (2*S*, 3*R*)	128	ND	ND	[46]
**39b** (2*R*, 3*S*)	50	ND	ND	
**21**	150	ND	ND	[48]
**22**	4	21 ^b^	ND	[45]
**41** (EGCG)	0.5	ND	ND	[49]
**42** (genistein)	30	>100	ND	[51]
**43** (nanaomycin A)	inactive	ND	0.5	[52]
**44** (SW155246)	1.2	38	ND	[53]

^a^ IC_50_ and EC_50_ (values in brackets) correspond to the half-maximal inhibitory concentration and the half-maximal effective concentration, respectively. Both are calculated from enzymatic assays. Assays are based either on the incorporation of radioactive methyl groups, or on methyl-sensitive restriction enzymes, or on the use of antibodies. ^b^ DNMT3a/3L complex. ND*:* Not Described.

**Table 2:** Examples of oligonucleotide-based inhibitors.

Entry	Inhibitor	Sequence (5’ to 3’)	IC_50_ (or *K_i_*) ^a^, μM			Reference
			(DNMT1)	(DNMT3a)	(DNMT3b)	
1	asCEBPα-2	GCCAGUGGCGAGGGGCGGCGCGG	(0.4341)	ND	ND	[55]
2	asCEBPα-2HPE	GACAGUGGAGAGGGGCGGAGCGG	(0.1352)	ND	ND	[55]
3	miR-155-5p	UUAAUGCUAAUCGUGAUAGGGGU	(0.02788)	ND	ND	[55]
4	MTC-423	CCTATGCGATCGAGTTTTCT[z]GAT[z]GCATAGG z = zebularine	0.363	1.60	17.5	[56]
5	MTC-427	CCTATG[M]GAT[M]GAGTTTTCT[z]GAT[z]GCATAGG′ M = methylcytosine; z = zebularine	0.295	1.52	6.20	[56]
6	MTC-433	CCTATG[M]GAT[M]GAGTTTTCT[dz]GAT[dz]GCATAGG M = 5-methylcytosine; dz = deoxyzebularine	0.00422	*ND*	*ND*	[56]
7	MG98	TTCATGTCAGCCAAGGCCAC	ND	ND	ND	[57,58]
8	miR29b	UAGCACCAUUUGAAAUCAGUGUU	ND	ND	ND	[59]

^a^ IC_50_ and *Ki* (values in brackets) correspond to the half-maximal inhibitory concentration and inhibition constant, respectively, calculated from enzymatic assays. ND*:* Not Described.

**Table 3:** HMT inhibitors and their activity.

Inhibitor	IC_50_^a^, µM						Reference
	Suv39H1	G9a	EZH2	DOT1L	CARM1 (PRMT4)	PRMT5	
**23** (BIX-01294)	>10	2.7	ND	ND	ND	ND	[81]
**24** (BIX-01338)	1.1	4.7	ND	ND	ND	ND	[81]
**25** (BRD9539)	ND	6.3	ND	ND	ND	ND	[83]
**27** (EPZ6438, tazemetostat, E7438)	ND	ND	0.012	>100	>100	>100	[115]
**28** (GSK2816126, GSK126)	>100	>100	0.009	>100	>100	>100	[90]
**29** (EPZ5676, pinometostat)	ND	ND	ND	0.0008	>50	30	[116]
**30** (CPI-169)	ND	ND	<0.001 ^b^	ND	ND	ND	[91]
**31** (EPZ004777)	ND	ND	>50 ^b^	0.0004	>50	0.521	[101]
**32 **	ND	ND	ND	0.0014	ND	ND	[111]
**33**	ND	ND	ND	0.0004	ND	ND	[111]
**34**	ND	ND	ND	0.014	ND	ND	[112]
**35** (ellagic acid, TBBD)	ND	ND	ND	ND	25	ND	[117]
**36** (GSK3235025, EPZ015666)	ND	ND	ND	ND	ND	0.022	[118]

^a^ IC_50_ corresponds to the half-maximal inhibitory concentration and they are calculated from enzymatic assays based on the use of radioactive AdoMet or on the use of antibodies. Suv39H1: Suppressor of variegation 3-9 homolog 1; G9a: euchromatic histone-lysine *N*-methyltransferase 2; EZH2: enhancer of zeste homolog 2; CARM1: coactivator-associated arginine methyltransferase.
